# Dissociating task difficulty from incongruence in face-voice emotion integration

**DOI:** 10.3389/fnhum.2013.00744

**Published:** 2013-11-13

**Authors:** Rebecca Watson, Marianne Latinus, Takao Noguchi, Oliver Garrod, Frances Crabbe, Pascal Belin

**Affiliations:** ^1^Department of Cognitive Neuroscience, Faculty of Psychology and Neuroscience, Maastricht UniversityMaastricht, Netherlands; ^2^Centre for Cognitive Neuroimaging, Institute of Neuroscience and Psychology, University of GlasgowGlasgow, UK; ^3^Institut de Neurosciences de la Timone and Aix-Marseille UniversitéMarseille, France; ^4^Department of Psychology, University of WarwickCoventry, UK; ^5^International Laboratories for Brain, Music and Sound, Université de Montréal and McGill UniversityMontreal, QC, Canada

**Keywords:** multisensory integration, emotion perception, functional magnetic resonance adaptation, incongruence, affective conflict

## Abstract

In the everyday environment, affective information is conveyed by both the face and the voice. Studies have demonstrated that a concurrently presented voice can alter the way that an emotional face expression is perceived, and vice versa, leading to emotional conflict if the information in the two modalities is mismatched. Additionally, evidence suggests that incongruence of emotional valence activates cerebral networks involved in conflict monitoring and resolution. However, it is currently unclear whether this is due to task difficulty—that incongruent stimuli are harder to categorize—or simply to the detection of mismatching information in the two modalities. The aim of the present fMRI study was to examine the neurophysiological correlates of processing incongruent emotional information, independent of task difficulty. Subjects were scanned while judging the emotion of face-voice affective stimuli. Both the face and voice were parametrically morphed between anger and happiness and then paired in all audiovisual combinations, resulting in stimuli each defined by two separate values: the degree of incongruence between the face and voice, and the degree of clarity of the combined face-voice information. Due to the specific morphing procedure utilized, we hypothesized that the clarity value, rather than incongruence value, would better reflect task difficulty. Behavioral data revealed that participants integrated face and voice affective information, and that the clarity, as opposed to incongruence value correlated with categorization difficulty. Cerebrally, incongruence was more associated with activity in the superior temporal region, which emerged after task difficulty had been accounted for. Overall, our results suggest that activation in the superior temporal region in response to incongruent information cannot be explained simply by task difficulty, and may rather be due to detection of mismatching information between the two modalities.

## Introduction

The recognition and understanding of emotion from the face and voice is a crucial part of social cognition and inter-personal relationships. In everyday life, however, the evaluation of emotion is rarely based on the expression of either of these modalities alone. Rather, we usually see a face whilst simultaneously hearing a voice, and combine this information to create a coherent, unified percept.

Neuroimaging studies of audiovisual emotion perception have typically compared the response to congruent audiovisual stimuli with purely auditory or visual ones. Regions that respond more to both, or each of the unimodal sources alone are assumed to play a part in integrating information from the two modalities. Studies using this approach have particularly emphasized the integrative role of the superior temporal gyrus (STG)/middle temporal gyrus (MTG) as well as the posterior STS (pSTS; Pourtois et al., 2005; Ethofer et al., 2006a; Kreifelts et al., 2007, 2009, 2010; Robins et al., 2009), amygdala (Dolan et al., 2001; Ethofer et al., 2006a,b) and insula Ethofer et al. (2006a); but also regions presumed to be part of the “visual” or “auditory” systems, such as the fusiform gyrus (Kreifelts et al., 2010) and anterior superior temporal gyrus (STG; Robins et al., 2009).

Another, less utilized approach has been to employ a congruence design where a condition with emotionally congruent bimodal stimulation is compared to emotionally incongruent bimodal cues (e.g., Dolan et al., 2001; Klasen et al., 2011; Müller et al., 2011). This comparison follows the assumption that only in a congruent condition can the unimodal inputs be truly integrated into a viable percept, and thus at the neural level regions responding more to congruent as opposed to incongruent information would be presumed to be involved in integrating multisensory information. Furthermore, a congruence design also allows the researcher to focus on the effects of affective conflict. Affective conflict can occur when information conveyed by the two modalities is not congruent, and we concurrently receive two or more different emotional inputs. Brain regions activated by affective conflict can be isolated by employing the reverse contrast; that is, incongruent vs. congruent information.

Non-emotional conflict has been studied extensively using both behavioral and neuroimaging experiments (e.g., Carter et al., 1998; MacDonald et al., 2000; Durston et al., 2003; Weissman et al., 2004). In contrast, very few studies have focused on the effects of affective conflict. Behaviorally, emotion conflict has been indicated by decreases in categorization accuracy and increased reaction times in incongruent compared to congruent conditions (de Gelder and Vroomen, 2000; Dolan et al., 2001; Collignon et al., 2008). Congruence effects have rarely been explored at the cerebral level, and studies which have done so have mainly focussed on isolating regions which integrate affective information, as opposed to those responding to emotional conflict.

For example, in an early study Dolan et al. (2001) compared activation in response to congruent and incongruent affective face-voice stimuli. They observed that there was an enhanced response in the left amygdala to congruent fearful stimuli (fearful voice + fearful face) compared with incongruent ones (happy voice + fearful face), suggesting that the amygdala is important for emotional crossmodal sensory convergence, specifically during fear processing. More recently, Klasen et al. (2011) investigated the multimodal representation of emotional information with dynamic stimuli expressing facial and vocal emotions congruently and incongruently. The authors observed that both congruent and incongruent audiovisual stimuli evoked larger responses in thalamus and superior temporal regions, compared with unimodal conditions, but that congruent emotions (compared to incongruent) elicited higher activation in the amygdala, insula, ventral posterior cingulate (vPCC), temporo-occipital, and auditory cortices. The vPCC exhibited differential reactions to congruency and incongruency for all emotion categories, and the amygdala for all but one, leading the authors to conclude that these may be regions specifically involved in integrating affective information from the face and the voice.

Recently, Müller et al. (2011) conducted a study which focussed on the neural correlates of audiovisual emotional incongruence processing. The authors scanned subjects as they judged emotional expressions in static faces while concurrently being exposed to emotional (scream, laughter) or neutral (yawning) sounds. The imaging data revealed that incongruence of emotional valence between faces and sounds led to increased activation in the middle cingulate cortex, right superior frontal cortex, right supplementary motor area as well as the right temporoparietal junction. These results correspond to those of Klasen et al. (2011), who observed that incongruent emotions (compared to congruent) activated a similar frontoparietal network and also the bilateral caudate nucleus. However, in contrast to the findings of Dolan et al. (2001), Klasen et al. (2011), Müller et al. (2011) reported that congruent compared to incongruent conditions did not evoke significantly increased activation in any brain region.

The limited, and on occasions conflicting evidence means that the effects of emotion incongruence still remain a relatively open question. Importantly, it should also be noted that the described studies confounded aspects of task difficulty with stimulus incongruence. Typically, the judgment of emotion is more difficult in an incongruent condition. Generally aspects of task difficulty are inherent to the task: emotional congruency facilitates emotion recognition, which is the major benefit of multimodal emotions. As such, congruent and incongruent trials are usually by definition characterized by differences in difficulty levels. However, this means that the neural correlates of task difficulty have still not been fully disentangled from the pure effects of emotional incongruency.

The purpose of the present study was to examine the neurophysiological correlates of processing incongruent emotional information, independent of task difficulty. A secondary aim was to search for regions specifically processing congruent stimuli, which could be presumed to be involved in multisensory integration. We parametrically morphed dynamic faces and voices between anger and happiness, and paired the resultant visual and auditory morphs to create a set of audiovisual stimuli that varied in not only the degree of incongruence between the face and voice, but also their presumed difficulty to classify. We assigned the audiovisual stimuli two values: one corresponding to the degree of incongruence between the face and the voice, and another corresponding to the degree of clarity in the combined face-voice information. Due to our use of morphing techniques, we hypothesized that the clarity value of a stimulus would be more related to task difficulty than its incongruence value, and that this distinction would allow us to separately examine the effects of incongruence and task difficulty. Participants were scanned in a rapid, efficiency-optimized event-related design [specifically, the continuous carry-over design (Aguirre, 2007)] while viewing the parametrically morphed audiovisual movies, and performing a 2-alternative forced choice emotion classification task. On the basis of previous results, we hypothesized that if activation in those networks associated with conflict monitoring was due to task difficulty, unclear as opposed to incongruent stimuli would provoke a response in these areas. We also hypothesized that once task difficulty was accounted for, stimulus incongruence might instead evoke responses in regions more associated with audiovisual (specifically, audiovisual affective) processing.

## Materials and methods

### Participants

Ten English-speaking participants [4 males and 6 females; mean age 27 years (SD ± 13 years)] took part in a pre-test of stimuli, in order to ensure there was appropriate categorization of unimodal emotion (see Appendix), and a new group of eighteen participants [10 males, 8 females, mean age: 25 years (SD ± 3.7 years)] were scanned in the main fMRI experiment. All had self-reported normal or corrected vision and hearing. The study was approved by University of Glasgow Ethics Committee and conformed to the Declaration of Helsinki. All volunteers provided informed written consent before, and received payment at the rate of £6 p/h for participation.

### Stimuli

#### Video recording

Two actors (one male, one female) participated in the video recording sessions. Both had studied drama at University level. The actors were paid for their participation at the rate of £6 p/h. Each actor sat in a recording booth, and was given instructions through an outside microphone connected to speakers within the booth. The actor wore a head cap, in order to hide the hair, and a marked head panel was fitted to the cap, which was used to determine head position. A Di3d capture system (see Winder et al., 2008) was used for the video recording. The actor sat between two camera pods, at a distance of 143 cm away from them both. Thus, each camera captured a slight side-view of the face, as opposed to a directly frontal view. Each pod consisted of a vertical arrangement of 3 different cameras. The top and bottom cameras were black and white, and were used to capture general shape information. The middle camera in each pod was a color camera, used to capture texture and color information. A lamp was placed behind each camera, and luminance kept constant at 21 amps. Video information was recorded by Di3D software on this PC as a series of jpegs at high resolution (2 megapixels). Vocal sound-information was transmitted via a Microtech Geffell GMBH UMT 800 microphone—positioned above the actor—to a second PC outside the booth, and was recorded at 44100 Hz using Adobe Audition (Adobe Systems Incorporated, San Jose, CA).

The actors were instructed to express anger and happiness in both the face and the voice. The sound “ah” was chosen as it contains no linguistic information. They were asked to sit as still as possible, in order to keep head movement to a minimum. Audiovisual expressions were produced a number of times, with a pause of three seconds between each repetition. The actor clapped in front of their face before they produced each set of expressions, which provided markers when later matching the audio recording to the video.

#### Video processing

Video output was split into a number of different sequences, where each sequence was made up of a number of jpegs (frames) and each repetition of each emotional expression formed one sequence. Two final sequences were chosen for each actor. Using the Di3D software, 43 landmarks were placed around the face and facial features in the first and last frame of the sequence, forming a landmark-mesh. An existing generic mesh was applied to the beginning and the end of the sequence (i.e., first and last jpeg), which was then warped to fit the landmark-mesh. The first mesh was then used to estimate the mesh position in the second jpeg, which was then used to estimate the position in the third and so on. This forward tracking/mesh estimation was then carried out in the opposite direction (i.e., the last mesh was used to estimate the mesh position in the jpeg before it). The two side-views of the actor, one from each camera pod, were merged together, forming one directly frontal view of the face. We smoothed the converging line, which ran from the forehead to the chin down the middle of the face, using average facial texture information. Any head movement was removed by tracking and aligning the eight marked points on the head panel, so that they were always in the same position throughout the sequence.

#### Audio processing

In addition to the original sound recording, a duplicate reduced-noise version was also produced. A recording made in the empty booth provided a “noise-baseline,” which was used to remove noise using a Fourier transform. The entire reduced-noise audio recording for each actor was then edited in Adobe Premiere (Adobe Systems Incorporated, San Jose, CA). Using the actor claps as markers for the start of each emotional expression, the audio sequences corresponding to the correct video sequence frames (at a frame rate of 25 frames per second) were identified and split into separate clips. The separate audio samples were then normalized for mean amplitude using Adobe Audition.

#### Video morphing

The video morphing was performed independently on the texture and shape components of the 4D models. The texture was warped onto a common template shape using the piecewise-affine warp and the morph was then performed as a weighted linear sum on the RGB pixel values; the shape was normalized for rigid head position (i.e., rotation, translation) using a combination of the ICP (Besl and McKay, 1992) and the RANSAC (Bolles and Fischler, 1981) methods and the morph was then performed as a weighted linear sum on the vertex coordinates. To account for timing differences between two expressions, pairs of matching anchor frames were selected in the two sequences corresponding to similar movement stages (for example, “mouth first opens,” “maximum mouth opening,” etc.). The sequence pairs were broken up into segment pairs between the anchor points and the lengths of the pixel and vertex time courses for the segment pairs were rescaled to be equal for the pair using linear interpolation. The new length was chosen as the average length of the segment over the pair. Finally, the segment pairs were reassembled into the full sequence pair and the morph was performed at each frame of the sequence. Five morph levels were chosen—ranging from 10 to 90% of one expression, in 20% steps—and the same morph level was used at each frame of the sequence, producing a total of five morph sequences which were rendered to video using 3DS Max.

#### Audio morphing

Auditory stimuli were edited using Adobe Audition 2.0. In order to generate the auditory components to the “morph-videos” three temporal and three frequency points were identified and landmarks corresponding to these set in the MATLAB-based morphing algorithm STRAIGHT (Kawahara, 2003), which were then used to generate a morph continuum between the two affective vocalizations equivalent to the faces. Two continua of voices—one for each actor, and consisting of five different voices ranging from 90% angry to 90% happy in 20% steps—were then generated by resynthesis based on a logarithmic interpolation of the angry and happy voices temporal and frequency anchor templates to a 50% average.

#### Audiovisual movie production

**Figure 1:**
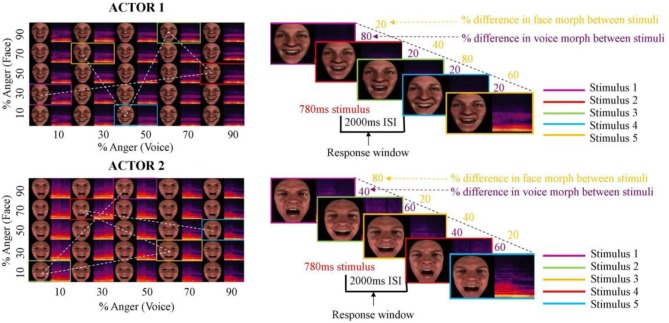
**Stimuli and continuous carry-over design**. Anger and happiness expressions produced by two actors were morphed in both face and voice between 10and 90% anger, in 20% steps. These were then paired in all possible combinations, creating 25 different audiovisual stimuli per actor. Left hand panels show face morphs and voice morphs, and resultant audiovisual pairings (examples in colored rectangles). Expressions from each actor were presented in two interleaved Type1 Index1 (*n* = 25) continuous carry-over sequences (sequential presentation indicated by dotted lines in left hand panels), over two experimental runs. Each block contained expressions from only one actor, and blocks were alternated between actor. Right hand panels indicate examples of within-block sequences of stimuli.

The auditory and visual morphing procedures produced five dynamic face videos and five audio samples for each actor. Within actor, these stimuli were all equal length. In order to ensure all stimuli were of equal length, we edited video and audio clips between actors. In all video clips, seven important temporal landmarks that best characterized the facial movements related to the vocal production were determined, and the frames at which they occurred were identified. These landmarks were the first movement of the chin, first opening of lips, maximum opening of the mouth, first movement of the lips inwards, time point at which the teeth met, closing of the lips, and the last movement of the chin. The theoretical average frames for these landmarks were then calculated, and the videos edited so the occurrence of these landmarks matched in all clips. Editing consisted of inserting or deleting video frames during fairly motionless periods. The editing produced ten adjusted video clips, each 18 frames (720 ms) long. The audio samples were then also adjusted in accordance with the temporal landmarks identified in the video clips, in order to create 10 vocalizations (5 for each actor) of equal length. Within actor, the five visual and five auditory clips were then paired together in all possible combinations. This resulted in a total of 25 audiovisual stimuli for each actor, parametrically varying in congruence between face and voice affective information (see Figure 1).

#### Definition of stimulus clarity and incongruence

Each stimulus was assigned “clarity” and “incongruence” values, which took into account the emotion morph of both the face and the voice. Incongruence was defined as the absolute (abs) value of face morph level minus voice morph level:

Incongruence=abs(% anger in face−% anger in voice)

Therefore, the higher values indicated the highest degree of incongruence.

However, we recognized that although completely congruent stimuli were all assigned the same value, some would presumably be easier to categorize than others (e.g., 90% angry face-90% angry voice as compared to 50% angry face-50% angry voice). Therefore, we took into account the clarity of the *combined* information of the face and the voice. To calculate a clarity value, we determined the average percentage of “anger” information contained in the stimulus. For example, the 90% angry face-90% angry voice stimulus contained 90% anger informativeness, and the 10% angry face-90% angry voice contained 50% anger informativeness—as did the 50% angry face-50% angry voice stimulus. Clarity was thus calculated:

$$\text{Clarity}=\text{abs}{\left[\text{50}\%-\left(\text{average }\%\text{ anger information}\right)\right]}^{*}\text{2}$$

**Figure 2:**
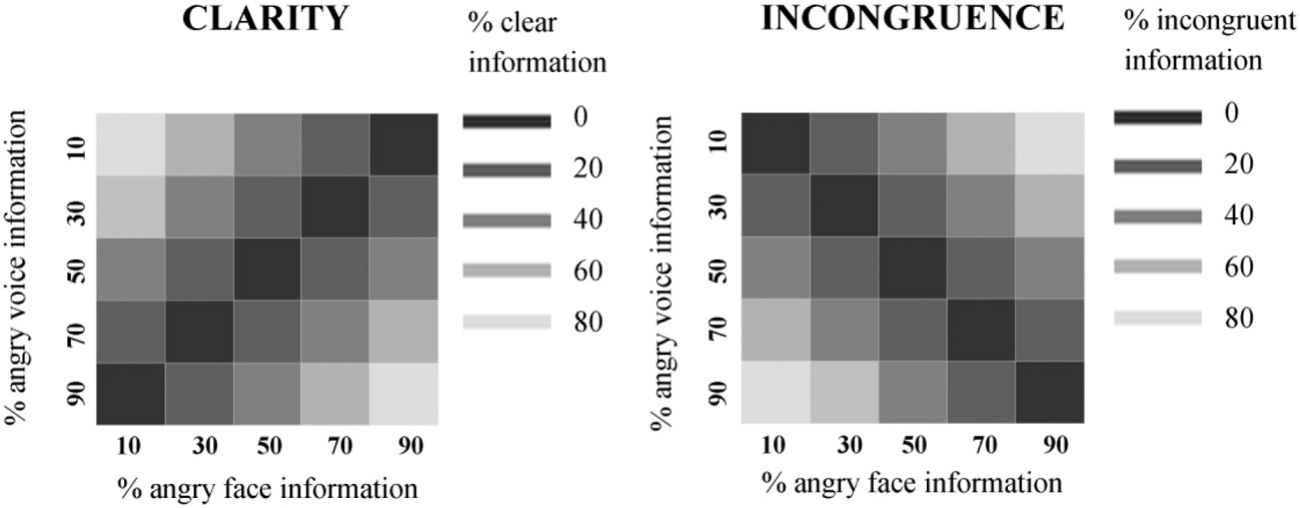
**Clarity and incongruence values assigned to face-voice stimuli. Left:** Clarity values. Each square in the grid represents one audiovisual stimulus. The horizontal value indicates the percentage of angry face information contained in the stimulus, and the vertical value represents the percentage of angry voice information contained in the stimulus. The color represents the clarity value of the stimulus (darkest = lowest clarity value = unclear combined information in the stimulus; lightest = highest clarity value = clear combined information in the stimulus). **Right**: Incongruence values. Each square in the grid represents one audiovisual stimulus. The horizontal value indicates the percentage of angry face information contained in the stimulus, and the vertical value represents the percentage of angry voice information contained in the stimulus. The color represents the incongruence value of the stimulus (darkest = lowest incongruence value = no difference in affect level of face and voice; lightest = highest = highest incongruence value = maximal difference in affect level of face and voice).

This resulted in clarity values which were a 90° rotation of our incongruence values in the 2D face-voice space (see Figure 2), where the values indicated the level of clear affective information contained within the stimulus as a whole. The higher values indicated a clearer combined emotion representation and lower values indicated an unclear combined emotion representation. For clarity and incongruence values assigned to each stimulus, refer to Figure 2. It should also be noted that there was a significant negative correlation between clarity and incongruency values (*r* = −0.556, *p* < 0.0001).

### Design and procedure

#### Continuous carry-over experiment

In the main experiment, stimuli were presented by way of a continuous carry over design (Aguirre, 2007). In summary, these are efficiency-optimized, serially balanced fMRI sequences in which every stimulus precedes and follows every other stimulus (i.e., the “Type 1 Index 1” sequence), which account for stimulus counterbalancing.

Carry-over designs are particularly useful when there is more complex variation in a set of stimuli, whose differences can be expressed as parametric changes along a number of different axes (e.g., face and voice emotion morph). A full description of the continuous carry over design can be found in Aguirre (2007). Stimuli were presented using the Psychtoolbox in Matlab, via electrostatic headphones (NordicNeuroLab, Norway) at a sound pressure level of 80 dB as measured using a Lutron Sl-4010 sound level meter. Before they were scanned, subjects were presented with sound samples to verify that the sound pressure level was comfortable and loud enough considering the scanner noise. Audiovisual movies were presented in two scanning runs (over two different days) while blood oxygenation-level dependent (BOLD) signal was measured in the fMRI scanner.

The stimulus order followed two interleaved *N* = 25 Type1 Index 1 sequences (one for each of the speaker continua; ISI: 2 s; Nonyane and Theobald, 2007), which shuffles stimuli within the continuum so that each stimulus is preceded by itself and every other within-continuum in a balanced manner. The sequence was interrupted by seven 20 s silent periods, which acted as a baseline, and at the end of a silent period the last 5 stimuli of the sequence preceding the silence were repeated before the sequence continued. These stimuli were removed in our later analysis. Participants were instructed to perform a 2 alternative forced choice emotion classification task (responses: Angry or Happy) using 2 buttons of an MR compatible response pad (NordicNeuroLab, Norway). They were also instructed to pay attention to both the face and voice, but could use the information presented in whatever way they wished to make their decision on emotion. Reaction times (relative to stimulus onset) were collected using Matlab with a response window limited to 2 s.

#### Imaging parameters

Functional images covering the whole brain (slices = 32, field of view = 210 × 210 mm, voxel size = 3 × 3 × 3 mm) were acquired on a 3T Tim Trio Scanner (Siemens) with a 12 channel head coil, using an echoplanar imaging (EPI) sequence (interleaved, *TR* = 2 s, *TE* = 30 ms, Flip Angle = 80°) were acquired in both the carry-over and localizer experiments. In total, we acquired 1560 EPI image volumes for the carry-over experiment, split into two scanning sessions consisting of 780 EPI volumes. The first 4 s of the functional run consisted of “dummy” gradient and radio frequency pulses to allow for steady state magnetization during which no stimuli were presented and no fMRI data collected. MRI was performed at the Centre for Cognitive Neuroimaging (CCNi) in Glasgow, UK.

At the end of each fMRI session, high-resolution T1-weighted structural images were collected in 192 axial slices and isotropic voxels (1 mm^3^; field of view: 256 × 256 mm^2^, *TR* = 1900 ms, *TE* = 2.92 ms, time to inversion = 900 ms, *FA* = 9°).

#### Imaging analysis

SPM8 software (Wellcome Department of Imaging Neuroscience, London, UK) was used to pre-process and analyse the imaging data. First the anatomical scan was AC-PC centered, and this correction applied to all EPI volumes.

Functional data were motion corrected using a spatial transformation which realigned all functional volumes to the first volume of the run and subsequently realigned the volumes to the mean volumes. The anatomical scan was co-registered to the mean volume and segmented. The anatomical and functional images were then normalized to the Montreal Neurological Institute (MNI) template using the parameters issued from the segmentation keeping the voxel resolution of the original scans (1 × 1 × 1 and 3 × 3 × 3, respectively). Functional images were then smoothed with a Gaussian function (8 mm FWHM).

**Figure 3:**
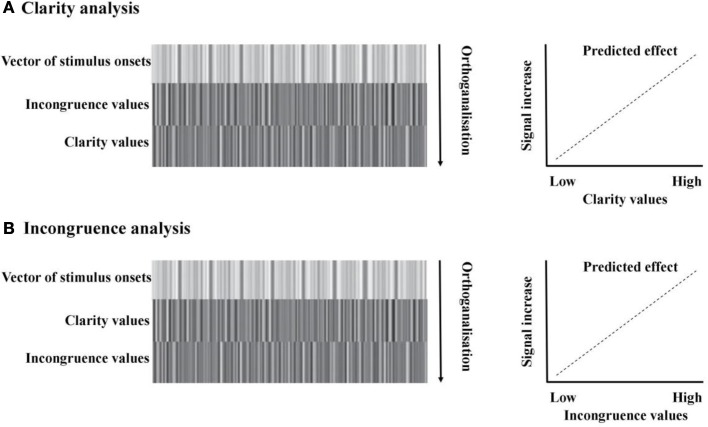
**fMRI analysis models. (A)** Clarity analysis. The design matrix included one vector modeling all the onsets of the audiovisual stimuli, then two parametric modulators: the first modeling the incongruence value of each stimulus, and the second modeling the clarity value of each stimulus. Parametric modulators were serially orthogonalized, meaning that any variance associated with incongruence was removed. The linear expansion of the parametric modulator predicted that, with a positive loading on the modulator, as clarity values increased, there would be a related increase in signal. **(B)** Incongruence analysis. The design matrix included one vector modeling all the onsets of the audiovisual stimuli, then two parametric modulators: the first modeling the clarity value of each stimulus, and the second modeling the incongruence value of each stimulus. As previously, parametric modulators were serially orthogonalized, meaning that any variance associated with clarity was removed. The linear expansion of the parametric modulator predicted that, with a positive loading on the modulator, as incongruence values increased, there would be a related increase in signal.

EPI time series were analyzed using the general linear model as implemented in SPM8. Functional data was further analyzed in two separate two-level random effects designs:

***Clarity.*** Brain activity time-locked to stimulus onset and duration was modeled against the 1st (linear) expansion of two parametric modulators: incongruence, then clarity. The second parametric modulator (clarity) was automatically orthogonalized with respect to the first (incongruence), meaning that any variance associated with incongruence was removed. The linear expansion allowed us to search for regions which showed a stepped, linear increase or decrease in signal in line with the linear increase/decrease in clarity of the audiovisual stimuli. This analysis is illustrated in (Figure 3A). The contrast for the effect of the second parametric modulator—clarity—was entered into separate second-level, group RFX analysis. We then further examined both positive and negative correlations of BOLD signal with clarity.

***Incongruence.*** Brain activity time-locked to stimulus onset was modeled against the 1st (linear) expansion of two parametric modulators: clarity, then incongruence. In contrast to the previous model, any variance associated with clarity was regressed out, isolating any effects due only to the degree of incongruence between the face and voice. This analysis is illustrated in (Figure 3B). The contrast for the effect of the second parametric modulator—incongruence—was entered into separate second-level, group RFX analyses. As in our clarity analysis, we then examined both positive and negative effects.

Reported results from the experimental run are from whole-brain analyses, masked by an experimental audiovisual vs. baseline contrast thresholded at *p* < 0.001 (voxel-level uncorrected), and are reported descriptively at a threshold of *p* < 0.05 (FWE voxel-level corrected).

## Results

### Behavioral data

#### Effects of face and voice morph

***Categorical data.*** Each participant's mean categorization values for each audiovisual emotion morph stimulus (collapsed across actor) was submitted to a two factor (face morph and voice morph), fully within subjects repeated measures ANOVA, with 5 levels per factor (percentage of “anger” information in the morph). This was in order to assess the overall contributions of face and voice emotion morph on categorical response.

The percentages of anger identification were of 96.3% (±4.7%) for the 90% angry face-90% angry voice stimulus and 2.78% (±3.59%) for the 90% happy face-90% happy voice stimulus. The percentage of anger identification for the 50% ambiguous angry-happy stimulus was 49.4% (±16.9%). The repeated measures ANOVA highlighted a main effect of voice morph [*F*_(1.14, 19.4)_ = 15.3, *p* < 0.002, η^2^_*p*_ = 0.473] and of face morph [*F*_(2.02, 34.3)_ = 348, *p* < 0.0001, η^2^_*p*_ = 0.953], and also a significant voice × face interaction [*F*_(5.78, 98.1)_ = 6.78, *p* < 0.0001, η^2^_*p*_ = 0.285].

*Post-hoc*, we compared categorization values between each of our completely congruent stimuli (i.e., 10% angry face-10% angry voice; 30% angry face-30% angry voice; 50% angry face-50% angry voice; 70% angry face-70% angry voice; 90% angry face-90% angry voice) in five paired *t*-tests. Each stimulus was compared to the next one (i.e., 10% angry face-10% angry voice vs. 30% angry face-30% angry voice; 30% angry face-30% angry voice vs. 50% angry face-50% angry voice and so on) to clarify whether each stimulus significantly differed from the other with regards to categorization. After a Bonferroni correction for multiple comparisons (level of significance: *p* < 0.01), we found each of the stimuli significantly differed from the next—10% angry face-10% angry voice vs. 30% angry face-30% [angry voice: *t*_(17)_ = −2.82, *p* < 0.0125; 30% angry face-30% angry voice vs. 50% angry face-50% angry voice: *t*_(17)_ = −13.7, *p* < 0.0001; 50% angry face-50% angry voice vs. 70% angry face-70% angry voice: *t*_(17)_ = −10.8, *p* < 0.0001; 70% angry face-70% angry voice vs. 90% angry face-90% angry voice: *t*_(17)_ = −5.44, *p* < 0.0001].

In a series of planned comparisons, we further examined at which points there were significant differences in categorization ratings between stimuli. We proposed that maximum incongruence between Face and Voice (i.e., 80% difference) would cause significant shifts in categorization, as compared to “end point” congruent stimuli (i.e., 10% angry face-10% angry voice; 90% angry face-90% angry voice). In order to test these hypotheses, we performed the following paired sample *t*-tests:

- 10% angry face-10% angry voice vs. 10% angry face-90% angry voice
- 10% angry face-90% angry voice vs. 90% angry face-90% angry voice
- 90% angry face-90% angry voice vs. 90% angry face-10% angry voice
- 90% angry face-10% angry voice vs. 10% angry face-10% angry voice

After a Bonferroni correction for multiple comparisons (level of significance: *p* < 0.0125), all comparisons were significant [*t*_(17)_ = −24.0, *p* < 0.0001; *t*_(17)_ = −3.42, *p* < 0.004; *t*_(17)_ = 27.6, *p* < 0.0001; *t*_(17)_ = 2.87, *p* < 0.0125, respectively].

**Figure 4:**
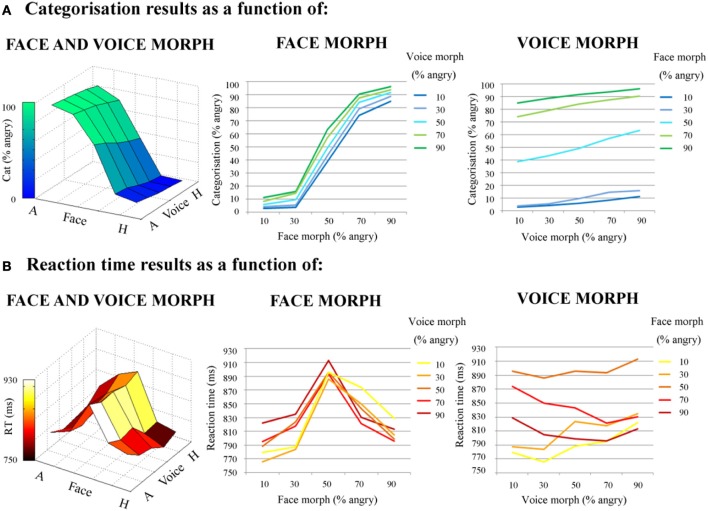
**Behavioral results; direct effects of face and voice emotion morph. (A)** Categorization results. Categorization (% angry responses) as a function of face morph (middle panel); voice morph (right panel) and both (left panel). **(B)** Reaction times results. Reaction time (ms) as a function of face morph (middle panel); voice morph (right panel) and both (left panel). Both face and voice morph were morphed between 10% happy and 90% happy, in 20% steps. Both categorization and reaction times results are averaged across actor. Note the greater influence of facial vs. vocal emotional cues on behavioral responses.

For an illustration of categorization results, refer to Figure 4.

***Reaction time data.*** Each participant's mean reaction time values for each stimulus (collapsed across actor) were firstly submitted to a two factor (face morph and voice morph), fully within subjects repeated measures ANOVA, with 5 levels per factor (percentage of “anger” information in the morph). As with categorical data, this was in order to assess the overall contribution of face and voice emotion morph—or the “direct effects” of face and voice morph—on reaction times.

The mean reaction times for the two end point congruent stimuli were 813 ms (±67.4 ms) and 779 ms (±64.4 ms) (for 10% angry face-10% angry voice and 90% angry face-90% angry voice, respectively). For the 50% angry face-50% angry voice stimulus the mean reaction time was 895 ms (±100 ms). Finally, for the two most incongruent stimuli (10% angry face-90% angry voice; 90% angry face-10% angry voice) these reaction times were 822 ms (±101 ms) and 829 ms (±92.5 ms). The ANOVA of reaction time data highlighted a main effect of voice morph [*F*_(2.91, 49.6)_ = 11.8, *p* < 0.0001, η^2^_*p*_ = 0.409] and of face morph [*F*_(2.34, 39.7)_ = 70.6, *p* < 0.0001, η^2^_*p*_ = 0.806], and also a significant interaction between the two modalities [*F*_(2.90, 39.4)_ = 7.40, *p* < 0.0001, η^2^_*p*_ = 0.303].

*Post-hoc*, we compared reaction time values between each of our completely congruent stimuli (i.e., 10% angry face-10% angry voice; 30% angry face-30% angry voice; 50% angry face-50% angry voice; 70% angry face-70% angry voice; 90% angry face-90% angry voice) in five paired *t*-tests. Each stimulus was compared to the next one (i.e., 10% angry face-10% angry voice vs. 30% angry face-30% angry voice; 30% angry face-30% angry voice vs. 50% angry face-50% angry voice and so on) to see whether each stimulus significantly differed from the other with regards to reaction time. After a Bonferroni correction for multiple comparisons (level of significance: *p* < 0.01), the following comparisons were significant—30% angry face-30% angry voice vs. 50% angry face-50% angry voice: *t*_(17)_ = −7.74, *p* < 0.0001; 50% angry face-50% angry voice vs. 70% angry face-70% angry voice: *t*_(17)_ = 7.67, *p* < 0.0001. The following comparisons were not significant—10% angry face-10% angry voice vs. 30% angry face-30% angry voice: *t*_(17)_ = −0.638, *p* = 0.532; 70% angry face-70% angry voice vs. 90% angry face-90% angry voice: *t*_(17)_ = 0.904, *p* = 0.379.

As in our categorization analysis, we proposed that maximum incongruence between Face and Voice (i.e., 80% difference) would take significantly longer to categorize, as compared to “end point” congruent stimuli. However, we also expected that some stimuli that were congruent, but with a lower clarity value (i.e., 50% angry face-50% angry voice), would take longer to categorize than end-point congruent stimuli. In order to test these hypotheses, we performed the following paired sample *t*-tests:

- 10% angry face-10% angry voice vs. 10% angry face-90% angry voice
- 10% angry face-90% angry voice vs. 90% angry face-90% angry voice
- 90% angry face-90% angry voice vs. 90% angry face-10% angry voice
- 90% angry face-10% angry voice vs. 10% angry face-10% angry voice
- 50% angry face-50% angry voice vs. 10% angry face-10% angry voice
- 50% angry face-50% angry voice vs. 90% angry face-90% angry voice

After a Bonferroni correction for multiple comparisons (level of significance: *p* < 0.0125), comparisons (i), (iv), (v), and (vi) were significant [*t*_(17)_ = −4.72, *p* < 0.0001; *t*_(17)_ = 3.25, *p* < 0.006; *t*_(17)_ = 10.67, *p* < 0.0001; *t*_(17)_ = 6.29, *p* < 0.0001, respectively], but comparisons (ii) and (iii) were not [*t*_(17)_ = 1.30, *p* = 0.210; *t*_(17)_ = −5.80, *p* = 0.569, respectively].

For an illustration of reaction time results, refer to Figure 4.

#### Effect of stimulus clarity and incongruence

We also computed a multiple regression analysis to investigate the relative contribution of stimulus and incongruence of our audiovisual stimulus on the reaction times in individual subjects. This analysis confirmed that clarity was significantly related to reaction time (β = −18.7, *t* = −4.43, *p* < 0.0001), with a lower level of clarity resulting in longer reaction times, but that incongruence was not (β = −7.78, *t* = −1.83, *p* = 0.067).

### fMRI results

#### Clarity

**Figure 5:**
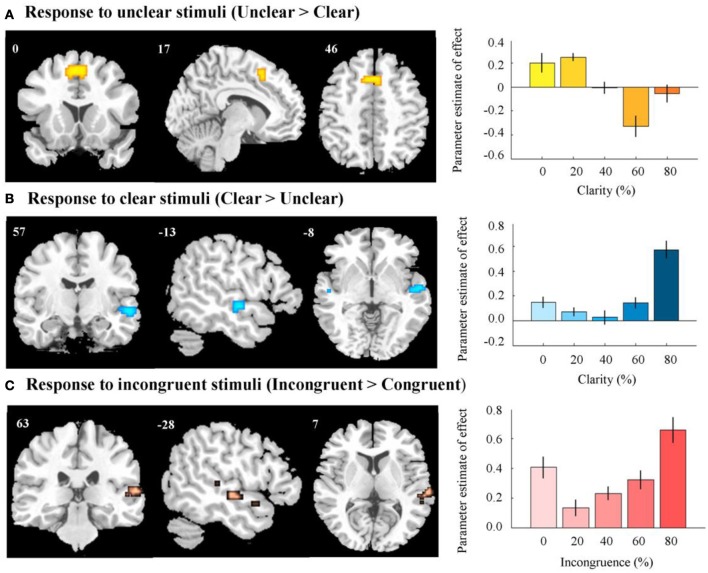
**fMRI results. (A)** Activation in anterior cingulate and supplementary motor area in response to unclear, compared to clear information. Right panel indicates response in peak activated voxel as a result of increasing level of clear information. **(B)** Activation in right STG/STS in response to clear, compared to unclear information. Right panel indicates response in peak activated voxel as a result of increasing level of clear information. **(C)** Activation in right STG/STS in response to incongruent, compared to congruent information. Right panel indicates response in peak activated voxel as a result of increasing level of incongruence. In all right panels, error bars represent the standard error of the parameter estimate of effect. Contrasts were thresholded to display voxels reaching a significance level of *p* < 0.05 (FWE voxel-corrected), and an additional minimum cluster size of greater than 5 contiguous voxels. Contrasts were masked by an AV vs. baseline contrast thresholded at *p* < 0.001 (uncorrected). MNI coordinates and t-scores are from the peak voxel of a cluster.

After removing the variance associated with incongruence, a positive effect of clarity was found in the right STG/superior temporal sulcus (Figure 5, Table 1A). These regions were more active when the audiovisual stimulus was clear. A negative effect was observed in the anterior cingulate gyrus, extending to the supplementary motor area—here, there was greater activation for the more unclear types of stimuli (Figure 5, Table 1B).

**Table 1:** **Effects of stimulus clarity and incongruence**.

**Brain regions**	**Coordinates (mm)**			***k***	***t*-statistic**
	***x***	***y***	***z***		
**(A) CLARITY (POSITIVE EFFECT; CLEAR > UNCLEAR)**					
Superior temporal gyrus/Superior temporal sulcus	57	−13	−8	35	9.14
**(B) CLARITY (NEGATIVE EFFECT; UNCLEAR > CLEAR)**					
Cingulate gyrus/Supplementary motor area	0	17	46	70	8.92
**(C) INCONGRUENCE (POSITIVE EFFECT; INCONGRUENT > CONGRUENT)**					
Superior temporal gyrus/Superior temporal sulcus	63	−28	7	25	6.96
Superior temporal gyrus/Superior temporal sulcus	54	−13	−8	24	6.60
**(D)INCONGRUENCE (NEGATIVE EFFECT; CONGRUENT > INCONGRUENT)**					
**NO SIGNIFICANT CLUSTERS**					

A,B. Positive and negative effects of clarity value of stimulus; C,D. Positive and negative effects of incongruence value of stimulus. Contrasts were thresholded to display voxels reaching a significance level of p < 0.05 (FWE voxel-corrected), and an additional minimum cluster size of greater than 5 contiguous voxels. Contrasts were masked by an AV vs. baseline contrast thresholded at p < 0.001 (uncorrected). MNI coordinates and t-scores are from the peak voxel of a cluster.

#### Incongruence

After the variance associated with clarity values was regressed out, we found a positive effect of incongruence across a wide region of the right STG/STS (Figure 5, Table 1C). This region appeared to respond more to incongruent information, as compared to congruent. We observed no negative effect of incongruence (i.e., congruent > incongruent), even at a relatively liberal threshold [*p* < 0.005 (voxel uncorrected)] (Table 1D).

## Discussion

In the present study we used visual and auditory morphing technologies to generate a range of face-voice stimuli parametrically varying in emotion, in conjunction with a continuous carry-over design so to examine the cerebral correlates of face-voice affect perception. Specifically, our main aim was to investigate the multimodal representation of emotion, and potential response to affective conflict, by observing the neural response to emotional incongruency in the face and voice. Furthermore, our intention was to investigate these effects independent of task difficulty, which has not yet been achieved in previous studies.

We firstly observed that emotion categorization, and speed of categorization, were modulated in line with parametric shifts in affective content of the face and voice: that is, the specific degree of morph of the face-voice stimulus had a direct effect on how angry or happy the participant viewed it, and as the information in the combined stimulus became increasingly unclear, the stimulus took longer to classify. Significantly, both modalities affected emotion perception—an integration effect—but face morph exerted a far larger influence on behavioral responses, both categorical and reaction times. This infers that participants found the faces in this study easier to categorize with regards to emotion as compared to voices. This is in line with other studies, where categorization has consistently been found to be more accurate and quicker for faces than to voices (e.g., Hess et al., 1988; de Gelder and Vroomen, 2000; Kreifelts et al., 2007; Collignon et al., 2008; Bänziger et al., 2009), although it should be noted that this will naturally vary dependent on the specific stimuli used from study to study.

We then investigated the effect of both stimulus clarity and incongruence on reaction time. Values for each of these dimensions were assigned based on where each stimulus lay in the 5 × 5 audiovisual emotion space: incongruence values related to the degree of discordance between the emotion displayed in the face and voice, whereas clarity values referred to how clear the affective information in the *combined* stimulus was.

We observed a significant effect of stimulus clarity on reaction time, with the more unclear stimuli taking longer to categorize. However, there was no significant effect of stimulus incongruence on reaction time. In similar studies it has been observed that generally, the greater the incongruence between face and voice, the more time it takes to classify the emotion (e.g., Massaro and Egan, 1996; de Gelder and Vroomen, 2000). However, due to the novel morphing procedure in our study some stimuli that were completely congruent would still have proved difficult for our participants to categorize—for example, those that had a pairing of ambiguous information in both the face and the voice. Thus, in this study it is unsurprising that the level of stimulus clarity was more reflective of task difficulty. This result meant we were able to take stimulus clarity as an indicator of task difficulty, and use these values to disentangle task difficulty from any observed incongruence effects.

At the cerebral level, we observed that there was an effect of both stimulus clarity and incongruence on brain activity. Firstly, we observed a negative effect of clarity in the anterior cingulate gyrus, extending to the supplemental motor area (SMA). In these latter regions, there was heightened activation in response to unclear stimuli (i.e., stimuli that were harder to categorize), as compared to clear stimuli.

In the study of non-emotional conflict, the cingulate gyrus [particularly, the anterior cingulate cortex (ACC)] is amongst the brain regions most frequently reported as being significantly activated when engaging in attentionally or behaviorally demanding cognitive tasks (Paus et al., 1998). A number of studies have also implicated this region in the detection of conflict between different possible responses to a stimulus, event, or situation (e.g., Carter et al., 1999; Kerns et al., 2004; Wendelken et al., 2009). The results of these studies have led to the conflict monitoring hypothesis, which suggests that conflict is detected by the dorsal ACC, which in turn recruits prefrontal regions to increase cognitive control (Botvinick et al., 2004; Kerns et al., 2004; Carter and van Veen, 2007). In our study, stimuli of an unclear or ambiguous nature were more demanding to categorize, requiring more energy for decision making, and thus it is unsurprising that we observed heightened activity in the cingulate in response to this information.

With regards to affective conflict, activation in ACC has been observed for (within-modality) conflicts in the visual and auditory domain (Haas et al., 2006; Ochsner et al., 2009; Wittfoth et al., 2010). Additionally, emotional conflict has been linked to the SMA, a region which plays a major role in voluntary action, cognitive control and initiation/inhibition of motor responses (Sumner et al., 2007; Grefkes et al., 2008; Kasess et al., 2008; Nachev et al., 2008). For example, SMA activation was found for emotional conflict in a study by Ochsner et al. (2009) in an affective flanker task, and in the previously cited study of Müller et al. (2011). These authors suggest that higher SMA activity may reflect increased executive control needed to select an adequate response in the presence of conflicting (emotional) stimuli.

Interestingly, incongruent stimuli did not elicit activation in these regions, as compared to congruent stimuli. As the clarity value of the stimulus was more linked to task difficulty than the incongruence value, we suggest that the cingulate and SMA respond specifically when there is difficulty in classifying a stimulus, as opposed to incongruence *per se*.

Instead, we observed a positive effect of incongruence across the bilateral STG/STS, in addition to a positive effect of clarity. Such regions have been implicated in auditory-visual processing and multisensory integration for both speech and non-speech stimuli (Calvert et al., 2000; Sekiyama et al., 2003; Beauchamp, 2005; Miller and D'Esposito, 2005). In overlapping regions, there was an increase in activation in response to stimuli that were by nature clear, and interestingly, also an increase in response to stimuli that were classified as incongruent.

With regards to incongruence, we might have expected that this pattern would be the reverse. One of the initial claims for the STS as an audiovisual binding site came from Calvert et al. (2000) who contrasted audiovisual speech to each modality in isolation (i.e., heard words or silent lip-reading). This revealed a super-additive response (i.e., a heightened response relative to the sum of the responses of audio and visual speech information presented alone) in the left pSTS when the audiovisual input was congruent but a sub-additive response when the audiovisual input was incongruent (i.e., showing a reduced response relative to the sum of the responses of audio and visual speech information presented alone). Moving from speech to emotion, Klasen et al. (2011) also found a stronger response to congruent vs. incongruent information in the amygdala and posterior cingulate, leading the authors to propose that these regions may be involved in integrating affective information. In contrast, we did not find any regions that responded more to congruent vs. incongruent information.

However, it should be noted that a number of studies have also produced conflicting results. Indeed, Hocking and Price (2008) stated that at that time they were unable to find any studies that replicated the Calvert et al. (2000) study showing enhanced pSTS activation for congruent relative to incongruent bimodal stimuli. In an fMRI study of the “McGurk effect”—a famous perceptual phenomenon observed in speech perception, where incompatible face-voice information leads to illusory percepts—conducted by Jones and Callan (2003), greater responses in the STS/STG for congruent audiovisual stimuli were not observed over incongruent audiovisual stimuli, as one might predict for a multisensory integration site. With regards to emotional incongruence, Müller et al. (2011) also did not observe a greater effect of congruent affective information over incongruent information in this region, or any others.

Hocking and Price (2008) suggest that potentially, one reason for the inconsistent congruency effects could be due to the fact that attention to one modality only during bimodal presentation elicits sub-additive effects (Talsma and Woldorff, 2005; Talsma et al., 2007). They argue that to minimize interference during incongruent audiovisual speech streams, participants may automatically or attentionally reduce visual processing (Deneve and Pouget, 2004; Ernst and Bulthoff, 2004), particularly in the study of Calvert et al. (2000) where congruent and incongruent conditions were presented in separate experiments with no instructions to attend to the visual stimuli. This would explain the absence of congruency effects in studies that presented brief stimuli or forced participants to attend to the visual input during incongruent audiovisual conditions.

Hocking and Price (2008) found that when task and stimulus presentation were controlled, a network of regions, including the pSTS, were activated more strongly for incongruent than congruent pairs of stimuli (stimuli were color photographs of objects, their written names, their auditory names and their associated environmental sounds). They suggest that activation reflects processing demand which is greater when two simultaneously presented stimuli refer to different concepts (as in the incongruent condition) than when two stimuli refer to the same object (the congruent condition). They also hypothesize that if participants were able to attend to one input modality whilst suppressing the other, then pSTS activation would be less for incongruent bimodal trials. In contrast, if subjects were forced to attend to both modalities then the pSTS activation would be higher for incongruent bimodal trials that effectively carry twice the information content as congruent trials.

In our study, a key point should be noted: values assigned to stimuli (specifically, those indicating incongruence) on the basis of the face and voice morph information were not necessarily reflective of the perceptual difficulty of classifying emotion. The incongruence value related to the degree of discordance between affect in the face and voice, whereas the clarity value related to how clear the overall, combined face-voice information was. Importantly, only clarity values were correlated with reaction times: the more unclear the combined information in the audiovisual stimulus was, the longer it took to classify. Although some incongruent stimuli resulted in shorted reaction times (e.g., 10% angry face-10% angry voice), some did not (i.e., 50% angry face-50% angry voice). Therefore, we can suggest that the heightened response to incongruent information across the right STS was not due to the perceptual difficulty of classifying the stimulus or processing demand.

In our study participants were instructed to attend both modalities: although we cannot be sure that participants definitely attended to both modalities in the incongruent trials, our behavioral data does suggest they did integrate the two modalities to some degree (indicated by a significant interaction between Face and Voice emotion morph for both categorical and reaction time data, in addition to a main effect of both modality). Therefore, in line with the proposal of Hocking and Price (2008), a tentative explanation is that participants were attending to both modalities and thus the STS activation was higher for incongruent bimodal trials. It is important to note that this is not necessarily reflective of greater perceptual difficulty in categorization (i.e., task difficulty). Rather, we propose this could be caused by the pure recognition that the auditory and visual inputs were different—an error detection.

Finally, Klasen et al. (2011) argue that incongruent emotional information cannot be successfully integrated into a bimodal emotional percept, and propose that regions responding more to congruent information than incongruent are reflective of an integrative process. However, Campanella and Belin (2007) suggested that conversely, it may be possible for incompatible affective information in the face and voice to be combined in such a way as to create an entirely new emotional percept, one independent of information contained in either modality—an “emotional McGurk effect.” This would imply some form of audiovisual integration, although perhaps one with a nature and mechanisms entirely different from the integration of emotionally congruent information. We are far from being able to conclusively answer this question; nonetheless, our results point to a strong activation in the STG/STS region in response to incongruent information that cannot be explained simply by task difficulty. We suggest that this instead could be due to an audiovisual mismatch detection, underlying the important role of the STG/STS in audiovisual processing.

## Conflict of interest statement

The authors declare that the research was conducted in the absence of any commercial or financial relationships that could be construed as a potential conflict of interest.

## Appendix

### Pre-test: stimulus validation

In a pre-test, using the separate group of ten participants, we investigated categorization of our stimuli across the two actors, firstly in order to ensure that expressions were recognized as intended, and secondly to clarify that there were no significant differences in categorization of expressions produced by different actors. Five participants were assigned to the expressions of the male actor, and another five were assigned to the expressions of the female actor. The stimuli were played to participants through a FLASH (www.adobe.com) object interface running on the Mozilla Firefox web browser. For each condition, stimuli were preloaded prior to running the experiment. The conditions were as follows:

#### Audio only

In this condition, participants heard a series of voices alone. They were instructed to listen to each voice, and make a forced choice decision on emotion based on the voice they had just heard, where the responses were either “Angry” or “Happy.” Again they indicated their decision via a button press. The five voice morphs were presented 10 times each, in a randomized order in one block consisting of 50 trials.

#### Video only

Participants saw all face videos, uncoupled with a voice. They were instructed to watch the screen and indicate their decision regarding emotion in the same way as before. The five faces were presented 10 times each, in randomized order in one block consisting of 50 trials.

Participants could respond either whilst the stimulus was playing, or after it ended. Regardless of when they responded, there was a 100ms wait until the next stimulus began playing. Conditions were counterbalanced between participants, with five participants for each of the two possible orders (collapsing across actor gender).

Categorization data was submitted to two, two factor mixed ANOVAs. In the first, degree of face emotion morph was a within subject factor, whilst the actor (male or female) was a between subject factor. This analysis highlighted a significant effect of face emotion morph on categorization [*F*_(1.35, 10.8)_ = 126, *p* < 0.0001]. There was no effect of actor on categorization [*F*_(1, 8)_ = 0.949, *p* = 0.359]. Face categorization results (averaged across actors) yielded the classic sigmoid-like psychometric function from the emotion classification task, with a steeper slope at central portions of the continua. The percentages of anger identification were 96% (±2.23%) for the 90% angry face, and 2% (±2.74%) for the 90% happy face. The 50% angry-happy face was identified as angry 53 times out of 100 (±9.75%). In the second ANOVA, degree of voice emotion morph was a within subject factor, whilst the actor was the between subject factor. This analysis highlighted a significant effect of voice emotion morph on categorization [*F*_(2.23, 17.7)_ = 127, *p* < 0.0001]. There was no effect of actor on categorization [*F*_(1, 8)_ = 0.949, *p* = 0.575]. Again, voice categorization results (averaged across actors) yielded the classis sigmoid-like psychometric function from the emotion classification task, with a steeper slope at central portions of the continua. The percentages of anger identification were 96% (±6.52%) for the 90% angry voice, and 0% (±0.00%) for the 90% happy voice. The 50% angry-happy voice was identified as angry 32 times out of 100 (±19.4%).
